# Varicella Zoster Virus and Relapsing Remitting Multiple Sclerosis

**DOI:** 10.1155/2011/214763

**Published:** 2011-03-30

**Authors:** Julio Sotelo, Teresa Corona

**Affiliations:** ^1^Neuroimmunology Unit, National Institute of Neurology and Neurosurgery of Mexico, Insurgentes Sur 3877, Mexico City 14269, Mexico; ^2^Clinical Laboratory of Neurodegenerative Diseases, National Institute of Neurology and Neurosurgery of Mexico, Insurgentes Sur 3877, Mexico City 14269, Mexico

## Abstract

Multiple sclerosis (MS) is an immune-mediated disorder; however, little is known about the triggering factors of the abnormal immune response. Different viruses from the herpes family have been mentioned as potential participants. Here, we review the evidences that support the association of varicella zoster virus (VZV) with MS. Epidemiological studies from geographical areas, where incidence of MS has increased in recent decades, pointed out a high frequency of varicella and zoster in the clinical antecedents of MS patients, and also laboratory investigations have found large quantities of DNA from VZV in leucocytes and cerebrospinal fluid of MS patients restricted to the ephemeral period of MS relapse, followed by disappearance of the virus during remission. The above observations and the peculiar features of VZV, mainly characterized by its neurotropism and long periods of latency followed by viral reactivation, support the idea on the participation of VZV in the etiology of MS. However, as with reports from studies with other viruses, particularly Epstein Barr virus, conflicting results on confirmatory studies about the presence of viral gene products in brain tissue indicate the need for further research on the potential participation of VZV in the etiology of MS.

## 1. Introduction

Several human pathogenic viruses have been, at one time or another, implicated as potential participants in the etiology of MS. Since the early 60s of the last century some studies indicated that, according to the clinical picture and the histopathological characteristics of MS lesions, a viral agent could be responsible for the disease [1]. Serological analysis of antiviral antibodies gave support to this hypothesis; in this way, some results suggested that viruses from the herpes family as well as other viruses from exanthematic diseases of childhood might be potential candidates [1–3]. However, most initial reports from positive studies disclosing viral DNA or antiviral antibodies could not be confirmed in subsequent investigations and were followed either by controversy or by novel results pointing out another viral candidate [4]. These failed attempts have been a common story for the last fifty years. It could be said that MS has been, over the decades, among the human diseases with most claims postulating etiological candidates; however, most corroborative studies have failed to replicate initial observations [2].

## 2. Autoimmunity versus Viral Infection in the Etiology of MS

Two main hypotheses have been constructed to explain the pathophysiology of MS: one is autoimmunity, the other an infectious agent, most probably a virus. In favor of the former a legion of studies has demonstrated the peculiar activation of the immune response during exacerbations of the disease. As the myelin is a highly antigenic structure capable of inciting an autoimmune response, it seems logical to postulate that MS might belong to the large group of autoimmune disorders. Although MS is obviously an immune-mediated disorder, some relevant obstacles exist to consider MS as a classical autoimmune disorder; among them is the lack of a replicative model of MS in experimental animals. This model, which should be identical to the human disease would result from the injection in healthy animals of the autologous antigen responsible for the autoimmune response, this requisite has been fulfilled in the case of other well-characterized autoimmune disorders of the nervous system like myasthenia gravis, experimental encephalitis (a model for post-vaccine encephalitis), and experimental polyneuritis (a model for Guillain-Barré Syndrome), but in the case of MS the absence of “experimental MS” has been replaced by “similar” but not identical experimental models [5, 6]. Another major obstacle to consider MS as a typical autoimmune disorder is the impossibility to transfer the disease from one affected individual to a healthy other by the injection of immune mediators such as immunoglobulins or immune cells, such as the case of disorders like myasthenia gravis or experimental encephalitis, where the injection either of IgG or T cells from a sick host to an unaffected one can translate temporarily the histopathological features of the disease. Additional evidence that challenges the autoimmune hypothesis of MS comes from recent reports that show the primary involvement of neural cells from grey matter and axons in the pathogenesis of MS in which axonal transection and neural injury are clearly evident in areas with normal-appearing white matter; these lesions in gray matter correlate with disabilities more strongly than white matter atrophy [7]. The primary lesions of neural cells rather than the unique participation of myelin antigens argues against the autoimmune hypothesis. Finally, the fact that the immune response is activated in restricted areas or plaques of the white matter, leaving unaffected many other sites containing the same myelin protein, is difficult to explain on the basis of an autoimmune etiology; if MS was of autoimmune origin, the same myelin protein everywhere in the brain would be involved and recognized by the immune activation; this is not the case in MS, where the immune-mediated lesion takes place within precise limiting borders amid an extensively myelinated zone, leaving unharmed neighboring areas [8].

In contrast with autoimmunity in the etiology of the disease the viral hypothesis would be more coherent with the pathophysiological process of MS; the segmentary lesions and the relapse-remission cycles do favor the idea of an infectious agent, most probably a virus involved as a triggering element of the immune reaction [9–11]. However, in comparison with the large body of evidence on the immune participation in the pathogenesis of MS the potential viral agent as the etiological cue has remained elusive [12]. 

In between the two possible etiologies of MS, autoimmunity and infection, the subject of genetic susceptibility has been explored recently with novel molecular techniques which have disclosed complicated genetic traits associated with MS [13–17].

## 3. Varicella Zoster Virus and Multiple Sclerosis

Herpes viruses hold very intriguing properties, among them is the capacity to remain latent in their host for long periods, even decades, and their ability to induce recurrent infections. Some herpes viruses are neurotrophic, particularly those from the subgroup alpha-herpes viruses, like herpes simplex virus and, most remarkably, varicella zoster virus (VZV) [18]. By means of the above properties these viruses remain within the nervous system from their hosts for decades producing periodic exacerbations; in the case of herpes simplex 1 the typical labial blisters, in the case of VZV the initial infection as chickenpox during childhood and late reactivations as zoster in older individuals. 

Although VZV has been postulated as a possible candidate to participate in MS [19–21], the results of most epidemiological or serological studies failed to confirm this link leaving uncertain the participation of VZV in the etiology of MS; a report evaluating 40 studies in the period 1965–1999 indicated that there is insufficient evidence to support the association of MS with VZV or zoster infections [22]. However, recent studies done in our laboratory began with the observation that the incidence of MS in Mexico was having a progressive increase, from a very rare disease in the 70s to a very frequent one nowadays, as seen by the growing incidence frequency of new cases in neurological wards throughout the country [23, 24]. If this was the case, the study of MS in Mexican patients represents a unique opportunity to search for the possible emergence of epidemiological factors related to the increase of exposure to novel environmental circumstances related with the etiology of MS. The initial hypothesis tested in MS patients was related to nutritional factors that have drastically changed during the past thirty years (adoption of foreign fast-food diets, changes in the scheme of traditional diets, introduction of industrialized foods) together with the investigation of individual exposure to factors that have been postulated as potential risks for MS such as personal pets, childhood infections, and previous allergies. Our results from that investigation pointed out as a sole highly significant risk factor for MS the antecedent of varicella during childhood [25, 26]. It seems important to stress the fact that this antecedent could only be uncovered in geographical areas where chickenpox is not a highly frequent disease of children [27, 28]. In this sense, global epidemiological studies have indicated that varicella is almost universal in cold and temperate countries from the northern hemisphere, whereas its incidence decreases drastically in tropical countries below the parallel 40, where Mexico is located [29]; in these areas the incidence of varicella during infancy is about fifty percent or lower. Coincidentally, the epidemiology of MS is similar to that of varicella, high in the northern hemisphere sharply decreasing towards tropical areas [30]. Our initial study demonstrated that the antecedent of varicella in MS patients was far more frequent than that in matched controls and in the general population [25, 31]; moreover, the mean age of infection in MS patients (8 years of age) was significantly older than in controls (5 years of age). The above unexpected findings prompted us to study the possible presence of VZV in MS patients: the initial study searched for DNA from VZV in peripheral mononuclear blood cells (PBMC) from 82 MS patients [32]; results showed that only 13 (16%) were positive by polymerase chain reaction (PCR) to one or various genes specific for VZV. However, when reviewing retrospectively the clinical charts from the few positive cases, to our surprise in all of them the blood samples had been taken within the first week of an acute relapse of MS, from this cohort only 2 patients with this particular condition were negative; thus, 11 MS patients studied during relapse (82%) were positive for VZV DNA. In contrast, all MS patients who were on remission at the time of the test were negative [32]. With these findings we hypothesized that the presence of VZV-DNA in PBMC, restricted to the initial days of an acute relapse, could signify either an epiphenomenon of virus reactivation due to the immune disturbances classically associated with episodes of MS exacerbation or that the VZV was primary involved in the etiopathogenesis of MS [32]. Subsequent studies have indicated that the latter explanation is indeed more feasible than the former. When PBMC from neurological patients with inflammatory, tumoral, or autoimmune ailments, as well as immunosuppressed patients were tested for VZV antigens, the results were all negative, identically to those obtained in healthy controls [33], thus showing that the abnormal activation or suppression of the immune response is not commonly associated with an epiphenomenon of VZV activation. It was also observed by real/time PCR that the amount of VZV found in PBMC from MS patients during relapse was high during the first week of the acute relapse but became gradually minor on clinical remission, until its disappearance in samples taken 2 months later [32–34]. These variations were observed in patients studied at various times during the cycle remission/relapse of MS. In these studies no similar phenomenon was seen when other herpes viruses were searched such as herpes simplex 1 and 2, Epstein Barr virus, human herpes virus 6, and cytomegalovirus [33, 35]. 

The above results strengthened the idea of a direct participation of VZV in the etiology of MS [36], not necessarily as casual infection but also possibly through host-viral immune interactions [37] which was corroborated in an additional study aimed to search for the virus within the central nervous system; the amount of viral particles from patients during relapse were measured simultaneously in PBMC and in CSF [38]. The amount of viral DNA measured in MS patients during relapse was hundreds of times higher in CSF than that in PBMC; in both locations, the amount decreased, as previously observed, after the acute relapse. Even more relevant, viral particles identical to VZV were observed by electron microscopy in the CSF from all 15 MS cases studied in relapse, these viral particles were precipitated with anti-VZV antibodies [38, 39]. Similar to the findings by PCR, the viral particles could not be observed in the CSF from the same MS patients when studied during remission [38].

## 4. Possible Pathway of VZV Infection in MS

VZV has the ability to remain latent in its host for long periods, which may extend several decades. If a subject suffers from varicella at 4 years of age and herpes zoster at 84 years of age, the virus remained viable, but in latency for an astonishing 80 years it is apparent that no other known viral disease infection reaches these extremes of latency, of which we know so little. From studies with subjects infected with HIV we already know that the same viral strain that caused the disease during childhood is the one that causes the disease in the elderly, giving support to the idea of a true lasting latency, rather than two episodes caused by nonrelated isolated infections separated by a very long period [18]. Also, the fact that both, varicella and zoster, are totally different diseases caused by the same infectious agent is intriguing and gives support to the concept of multiple viral pathogenicity [40], where the same virus can produce various diseases according to the age and susceptibility of the subject. Thus, it might be postulated that VZV produces varicella, a systemic disease at early ages of the host; it also produces zoster, a local disease of the peripheral nervous system in the elderly, and we speculate, based on our findings, that it could also produce MS, a local disease of the central nervous system during adulthood. In the case of varicella the viral disease behaves as a highly infectious and contagious infection associated with low pathogenicity [28], whereas in the case of zoster and MS it behaves as a local infection either of the peripheral or the central nervous system, the former as a centrifugal and the latter as a centripetal spread, from the neural ganglia hosting the latent virus; the development of these disorders would largely depend on individual susceptibility within the still enigmatic process of viral latency and reactivation [9, 41]. The hypothetical etiological mechanism of VZV infection in MS could be the sudden activation of a latent VZV in the neural ganglia which would spread to the central nervous system, perhaps through the ependymal cells, gaining access to the brain or the spinal cord via the ventricles and the central canal. The usual lesions located mostly in periventricular areas, as seen on imaging studies from MS patients, support this idea.

Although various herpes viruses have been implicated as participants in the etiology of MS, the most cited by current studies are human herpes virus 6 and Epstein Barr virus [2, 12, 42]. However, these two candidates have been challenged by other investigations [35, 43]. In our studies, findings of DNA from these two viruses in MS patients were not different from controls; also, particularly during exacerbations of MS the results were not different from those obtained during remission. The participation of VZV in the etiopathogenesis of MS still has to be corroborated by additional studies; so far, two investigations have confirmed our initial reports [32, 33, 38] on the presence of VZV in MS [44, 45]; in contrast, Burgoon et al. failed to show the presence of VZV virions or DNA in the CSF or in the acute plaques of MS patients, whereas recombinant antibodies prepared from clonally expanded plasma cells in MS CSF, which are thought to represent the intrathecally synthesized oligoclonal IgG, did not bind to VZV-infected cells [46]. Nevertheless, recent results from our laboratory have also documented the presence of VZV DNA in progressive forms of MS but in minor quantity from that found in CSF or in blood samples taken during MS relapse in patients with the relapse/remission form of MS [47]. Similar to these controversial results on VZV, in the case of EBV, another herpes virus that has been implicated by many recent reports as potential participant in the etiology of MS [48–52], various studies have failed to replicate initial results [53–57], but it has been demonstrated that a history of infectious mononucleosis is associated with an increased risk to develop MS [58]. These arguments on viral candidates possibly implicated in the etiology of MS indicate the need for original investigative approaches either to confirm or to discard definitively the participation of these or any other virus in the etiopathogenesis of MS.

 If VZV has a relevant role in the etiology of MS, several questions, related to individual genetic susceptibility and to the mechanisms of viral latency and reactivation of VZV from the dorsal root ganglia towards the CNS, are interesting challenges for future research.
